# Molecular mechanism of lncRNA SNHG12 in immune escape of non-small cell lung cancer through the HuR/PD-L1/USP8 axis

**DOI:** 10.1186/s11658-022-00343-7

**Published:** 2022-06-03

**Authors:** Yusheng Huang, Lei Xia, Xiangwu Tan, Jingyi Zhang, Weiwei Zeng, Benxu Tan, Xian Yu, Wei Fang, Zhenzhou Yang

**Affiliations:** 1 Department of Cancer Center, The Second Affiliated Hospital of Chongqing Medical University, Tianwen Avenue No. 288, Nan’an District, Chongqing, 400010 China; 2 Chongqing University, Three Gorges Hospital, No. 165 Xincheng Road, Wanzhou District, Chongqing, 404100 China

**Keywords:** Non-small cell lung cancer, LncRNA SNHG12, PD-L1, USP8, Immune escape, HuR, Peripheral blood mononuclear cells, CD8^+^ T

## Abstract

**Background:**

The pivotal role of long noncoding RNAs (lncRNAs) in cancer immune responses has been well established. This study was conducted with the aim of exploring the molecular mechanism of lncRNA small nucleolar RNA host gene 12 (SNHG12) in immune escape of non-small cell lung cancer (NSCLC).

**Methods:**

Expression of lncRNA SNHG12, programmed cell death receptor ligand 1 (PD-L1), ubiquitin-specific protease 8 (USP8), and human antigen R (HuR) in NSCLC tissues and cells was measured, and their binding relationship was determined. NSCLC cell proliferation and apoptosis were assessed. Peripheral blood mononuclear cells (PBMCs) were co-cultured with NSCLC cells. The ratio of CD8^+^ T cells, PBMC proliferation, and inflammatory factors were determined. lncRNA SNHG12 localization was assessed via subcellular fractionation assay. The half-life period of mRNA was determined using actinomycin D. Xenograft tumor models were established to confirm the role of lncRNA SNHG12 in vivo.

**Results:**

LncRNA SNHG12 was found to be prominently expressed in NSCLC tissues and cells, which was associated with a poor prognosis. Silencing lncRNA SNHG12 resulted in the reduction in proliferation and the promotion of apoptosis of NSCLC cells, while simultaneously increasing PBMC proliferation and the ratio of CD8^+^ T cells. Mechanically, the binding of lncRNA SNHG12 to HuR improved mRNA stability and expression of PD-L1 and USP8, and USP8-mediated deubiquitination stabilized the protein level of PD-L1. Overexpression of USP8 or PD-L1 weakened the inhibition of silencing lncRNA SNHG12 on the immune escape of NSCLC. Silencing lncRNA SNHG12 restricted tumor growth and upregulated the ratio of CD8^+^ T cells by decreasing USP8 and PD-L1.

**Conclusion:**

LncRNA SNHG12 facilitated the immune escape of NSCLC by binding to HuR and increasing PD-L1 and USP8 levels.

## Background

Non-small cell lung cancer (NSCLC) is the most common form of lung cancer (LC) (approximately 85% of all LC cases), consisting of three clinical subtypes: adenocarcinoma, squamous cell carcinoma, and large cell cancer [1]. In recent years, the discovery of some immune checkpoint blockers (ICBs) targeting lung tumors has demonstrated the immunogenic character of NSCLC; these findings have been determined to have survival benefits for patients with NSCLC [2]. For instance, the survival year was shown to be extended by 1 year in patients with NSCLC who have been treated with nivolumab compared with those treated with traditional platinum-based regimens [3]. Although ICBs are widely applied for the treatment of advanced NSCLC, most patients experience inherent or acquired resistance during immunotherapy [4]. Inherently, the tumor microenvironment of NSCLC allows for the potent activation of immunosuppressive cells, which are responsible for the secretion of immunosuppressive molecules or their receptors, thus establishing cancer resistance to ICBs [5]. Moreover, with the advancement in biotechnology, modulating cell functions at the molecular level provides novel insight into the treatment of NSCLC [6, 7]. As a result, it is important to identify novel molecules that could potentially reverse or prevent immune escape, which will improve the prognosis and survival of patients with NSCLC.

Long noncoding RNAs (lncRNAs), molecules longer than 200 nucleotides, exhibit expression alterations and mutations in the tumor microenvironment and harbor pro-/antitumor properties [8]. Some lncRNAs play an increasing role in the onset and development of NSCLC [9–11]. One such lncRNA, small nucleolar RNA host gene 12 (SNHG12), is ectopically expressed in a variety of malignancies and protects cancer cells against immune attack by crosstalk with the polarization of effector immune cells, including macrophages and T cells [12]. In particular, patients with NSCLC have been found to have enriched lncRNA SNHG12, which contributes to NSCLC cell growth, tumor metastasis, and drug resistance [13–15]. However, its role in the immune escape of NSCLC remains unknown and warrants further exploration.

Moreover, lncRNA SNHG12 has been shown to be present in the cytoplasm, thereby binding to cytoplasmic RNA‐binding proteins and further affecting expression of downstream protein targets [16]. Preexisting literature has shown that lncRNA SNHG12 can bind to human antigen R (HuR) in glioma and gastric cancer [17, 18]. HuR has been identified as an oncoprotein that activates cancer phenotypes of downstream mRNA-encoded proteins [19]. Programmed death-ligand 1/programmed death-1 (PD-L1/PD-1) is identified as an immune checkpoint in cancers that blocks the activation of immune cells and the generation of antibodies and cytokines, resulting in immune escape of tumors [20]. In NSCLC, HuR increases the mRNA stability of PD-L1, enhancing the immunosuppressive capacity of tumors [21]. Meanwhile, HuR increases mRNA stability and protein level of ubiquitin-specific protease 8 (USP8) as a result of its posttranscriptional regulation, which was determined to be the case in bladder cancer [22]. Loss of USP8 leads to the subsequent suppression of proliferation, migration, and invasion, while inducing apoptosis of NSCLC cells [23, 24]. In addition, as a deubiquitinase, USP8 improves the stability of oncoproteins by preventing ubiquitin-dependent degradation [25, 26]. Yet, whether USP8 plays a regulatory role in PD-L1 expression and immune escape in NSCLC remains unclear and requires further investigation.

Overall, the aforementioned findings indicate that lncRNA SNHG12 may be capable of increasing mRNA stability and expression of PD-L1 and USP8 via binding to HuR, thus contributing to immune escape of NSCLC. Consequently, the current study was conducted with the main objective of providing a novel theoretical reference for the application of SNHG12 in the immunotherapy of NSCLC.

## Materials and methods

### Collection of clinical samples

This study was approved by the Medical Ethics Committee of The Second Affiliated Hospital of Chongqing Medical University ((2022)624) (16 March 2022) and followed the Declaration of Helsinki. A total of 65 pairs of NSCLC tissue and adjacent normal tissue samples were collected from patients with NSCLC (31 males, 34 females, with an average age of 55.89 years) who received surgery, without any exposure to radiation or chemotherapy. Written informed consent was obtained from all participating patients. All patients were diagnosed histologically and independently by two experienced pathologists. Peripheral blood samples were collected from all patients 1 month before surgery. Tissue specimens were stored in liquid nitrogen at −80 °C for further analysis. Clinicopathologic features and the overall survival rate of patients with a survival of over 5 years were recorded in detail.

### Cell culture

Four LC cell lines (A549, SW1573, H1975, and H1299) and one normal bronchial epithelial cell line (BEAS-2B) were provided by ATCC (Manassas, VA, USA) and cultured in Roswell Park Memorial Institute (RPMI)-1640 medium (Thermo Fisher Scientific, Waltham, MA, USA) containing 10% fetal bovine serum (FBS) (YuBiotech, Shanghai, China) and 1% antibiotic solution (Leagene, Haidian, Beijing, China) at 37 °C with 5% CO_2_.

### Cell treatment

SNHG12 siRNA (si-SNHG12#1, si-SNHG12#2, and si-SNHG12#3), HuR siRNA (si-HuR#1, si-HuR#2, and si-HuR#3), USP8 siRNA (si-USP8#1, si-USP8#2, and si-USP8#3), and corresponding negative controls (si-NC), USP8 pcDNA3.1 (pc-USP8), PD-L1 pcDNA3.1 (pc-PD-L1), and corresponding negative controls (pc-NC) were all provided by Shanghai GenePharma Co., Ltd. (Shanghai, China). Following the manufacturer’s instructions, the above siRNAs or plasmids were transfected into A549 or H1299 cells using Lipofectamine 2000 kit. Cells of the third and fourth generations were used in experiments.

### Cell Counting Kit-8 (CCK-8) assay

The proliferation potential of NSCLC cells was evaluated using the CCK-8 (Beyotime, Haimen, Jiangsu, China). Briefly, transfected A549 or H1299 cells were collected and seeded into 96-well plates (3 × 10^3^ cells per well) and kept at 37 °C with 5% CO_2_. On days 0, 1, 2, and 3, 10 μL CCK-8 solution was added to each well for 2-h incubation at 37 °C. The absorbance was determined at a wavelength of 450 nm using a Labserv K3 microplate reader (WoYuan, Honghou, Shanghai, China).

### Colony formation assay

For colony formation assay, transfected A549 or H1299 cells (*N* = 500) underwent seeding into six-well plates and were cultured with RPMI 1640 medium in each well at 37 °C with 5% CO_2_ for 14 consecutive days. The cloned spots were fixed with 4% formaldehyde (Bogoo, Shanghai, China) for 15 min, stained with 0.1% crystal violet (Solarbio, Beijing, China) for 5 min, and washed with phosphate-buffered saline (PBS). Afterwards, colonies composed of up to 50 cells were counted under an EVOS M5000 microscope (Thermo Fisher Scientific).

### Isolation of peripheral blood mononuclear cells

Peripheral venous blood (2 mL) was collected from each patient and loaded into vials supplemented with ethylenediaminetetraacetic acid as the anticoagulant. According to the provided instructions, peripheral blood mononuclear cells (PBMCs) were isolated using the Ficoll density separation method (Tianjin Hao Yang Biological Manufacture Co., Ltd, Tianjin, China) and kept at −80 °C for subsequent experimentation. While waiting for analysis, PBMCs were cultured in RPIM-1640 medium containing 20% heat-inactivated FBS (GE Healthcare, Little Chalfont, UK) and 10% dimethylsulfoxide (Sigma-Aldrich, St. Louis, MO, USA). PBMCs of the third and fourth generations were used for the subsequent experiments.

### Co-culture and lactate dehydrogenase (LDH) cytotoxicity assays

PBMCs were cultured in RPMI-1640 medium and activated by Dynabeads Human T-Activator CD3/CD28 (Gibco, Grand Island, NY, USA) for 1 week. A549 or H1299 cells were seeded into 12-well plates at the cell-dependent concentration. After 24 h, activated PBMCs were co-cultured with adherent NSCLC cells at a ratio of 3:1 for 48 h. After co-culture, cell debris was removed and PBMCs were harvested with the application of the density separation method. PBMC cytotoxicity was detected using the LDH cytotoxicity detection kit (Thermo Fisher Scientific).

### Flow cytometry

Cell apoptosis was assessed using annexin V–fluorescein isothiocyanate (FITC)/propidium iodide (PI) apoptosis detection kit (Beyotime). Transfected A549 or H1299 cells were collected at a concentration of 2 × 10^5^ cells per well, received treatment with trypsin, and underwent resuspension with 400 μL PBS. Next, the cells were stained with 5 μL annexin V–FITC and 5 μL PI at room temperature under dark conditions for 15 min. After washing, the cells were analyzed with the use of a FACScan flow cytometer (BD Biosciences, San Jose, CA, USA).

PBMCs that had been collected were fixed and permeated using BD Cytofix/Cytoperm kit (BD Biosciences). Following the standard procedures of flow cytometry, cells were stained with anti-CD3 (ab16669, Abcam, Cambridge, MA, USA), anti-CD45 (ab40763, Abcam), or anti-CD8 (ab237709, Abcam). The results were obtained from the Fortessa platform (BD Biosciences) and analyzed using FlowJo software (BD Biosciences). With CD45 expression, size, and granularity (forward scatter versus side scatter) serving as references for gating, CD8^+^ T cells were further analyzed using CD3 and CD8.

### PBMC proliferation assay

PBMCs were fixed with 4% paraformaldehyde, permeated with 0.1% Triton X-100, and blocked with 3% bovine serum albumin. As for Ki-67 staining, cells underwent incubation with the primary antibody anti-Ki-67 (ab15580, Abcam) at 4 °C overnight and then with the secondary antibody IgG (Alexa Fluor 647) (ab150075, Abcam). Nuclei were counterstained with 4′,6-diamidino-2-phenylindole (Sigma-Aldrich). After sealing with the sealing solution supplemented by antifluorescence quenching agent, slides were photographed and analyzed by a fluorescence microscope (Olympus, Tokyo, Japan) and ImageJ.

### Enzyme-linked immunosorbent assay

Under the manufacturer’s instructions, the levels of interferon-gamma (IFN-γ) (ab174443), tumor necrosis factor (TNF)-α, transforming growth factor (TGF)-β1(ab100647), and interleukin (IL)-10 (ab185986) were measured in the supernatant of co-cultured PBMCs using commercially available ELISA kits (Abcam).

### Subcellular fractionation assay

The nuclear and cytoplasmic components were separated from A549 or H1299 cells using PARIS kits (Life Technologies, Carlsbad, CA, USA). Briefly, 2 × 10^6^ cells were collected in a centrifuge tube and underwent two washes with icy PBS. Then, 450 μL cell fractionation buffer (from the kits) was added. Subsequently, the compound was incubated on ice for 5 min, after which centrifugation was carried out at 500*g* and 4 °C for 5 min. Then, cytoplasmic RNA was collected from the supernatant, and nuclear RNA was collected from the pellets. U6 and GAPDH served as the control for the nucleus and cytoplasm, respectively.

### Bioinformatics analysis

The expression pattern of SNHG12 in NSCLC was analyzed with the use of starBase (https://starbase.sysu.edu.cn/panCancer.php) [27]. The association between SNHG12 expression and the prognosis of patients with NSCLC was predicted with the help of the GEPIA database (http://gepia.cancer-pku.cn/) [28]. The scores of SNHG12 binding to HuR, and HuR binding to PD-L1 and USP8 were predicted using RNA–Protein Interaction Prediction (RPISeq) (http://pridb.gdcb.iastate.edu/RPISeq/) [29].

### RNA immunoprecipitation (RIP) assay

RIP assay was conducted in A549 or H1299 cells using Magna RIP kits (Millipore, Billerica, MA, USA) to detect interactions between SNHG12 and HuR as well as HuR and PD-L1/USP8. After lysis in the RIP lysis buffer, the 100 μL whole-cell extract was incubated with the RIP lysis buffer containing anti-argonaute 2 (Ago2) (ab186733, Abcam)/anti-HuR (ab200342, Abcam)/anti-IgG (ab172730, Abcam)-conjugated magnetic beads. Proteins in precipitates were incubated with protease K buffer (Sigma-Aldrich) to separate immunoprecipitated RNA, which was followed by RT-qPCR.

### RNA stability

A549 or H1299 cells were exposed to actinomycin D (10 μg/mL) for 0, 4, 8, 12, and 24 h. Afterwards, total RNA was collected from cells, and PD-L1 or USP8 expression was measured with RT-qPCR.

### Co-immunoprecipitation (Co-IP)

After undergoing lysis in radio immunoprecipitation assay (RIPA) buffer (Thermo Fisher Scientific) at 4 °C for 30 min, A549 or H1299 cells were centrifuged at 4 °C and 1300*g* for 30 min. Subsequently, the supernatant was incubated with 1 μg corresponding antibodies (USP8, ab228572; IgG: ab133470; Abcam) at 4 °C overnight, after which incubation was carried out with Pierce protein A/G magnetic beads (Thermo Fisher Scientific) at 4 °C for 4 h. After 3-min centrifugation at 4 °C and 3000*g*, beads were washed and mixed with the loading buffer, followed by sodium dodecyl sulfate–polyacrylamide gel electrophoresis (SDS-PAGE) and western blot.

### PD-L1 ubiquitination detection

A549 or H1299 cells or tumor tissues in different treatment groups were lysed in RIPA lysis buffer. The lysis solution was harvested and incubated with IgG (ab133470, Abcam) or PD-L1 (ab213524, Abcam) and protein A/G agarose (Novex, Oslo, Norway) at 4 °C overnight. Subsequently, the cells were added with ubiquitination antibody (Ub) (ab19247, Abcam), after which western blot was performed.

### Animal modeling and treatment

All animal experiments were approved by the Ethics Committee of The Second Affiliated Hospital of Chongqing Medical University and were carried out in line with the Guide for the Care and Use of Laboratory Animals provided by National Institutes of Health [30] and the Basel Declaration. C57BL/6 mice [Guangzhou Institutes of Biomedicine and Health, Guangzhou, China, SYXK (Guangdong) 2018-0131] were housed under specific pathogen-free conditions, with free access to food and water. A549 cells were infected by lentiviral interfering vectors containing SNHG12 shRNA (sh-SNHG12) or NC shRNA (sh-NC) (Thermo Fisher Scientific), while cells with stable expression were screened by puromycin. Each mouse was subcutaneously injected with the stably expressed cells on the right flank with 3 × 10^5^ A549 cells. On day 7, tumor volume was measured using a caliper and calculated using the formula (length × width^2^)/2 [31]. On day 21, mice were euthanized with an injection of 150 mg/kg pentobarbital sodium via caudal veins. Afterwards, tumors were removed and weighed. A portion of tumor tissues was used for immunohistochemical analysis, and the remainder was used for reverse-transcription quantitative polymerase chain reaction (RT-qPCR) and ubiquitination detection.

### Immunohistochemistry

Tissue sections with a thickness of 5 μm were deparaffinized with xylenol for 20 min, rehydrated with gradient ethyl alcohol (100%, 90%, and 70%) for 5 min, washed with running water, boiled in 10 mM citrate buffer for 15 min, and incubated with PBS containing 5% H_2_O_2_. After blockade with 10% FBS, sections were incubated with anti-Ki67 (ab15580, Abcam), ani-PD-L1 (ab205921, Abcam), anti-USP8 (ab228572, Abcam), and anti-CD8 (ab237709, Abcam) at 4 °C overnight for immunohistochemical staining. Subsequently, incubation was carried out on the sections with the addition of biotinylated secondary antibody (ab205718, Abcam) at room temperature for 1 h. Following staining with diaminobenzidine for 5 min, sections were rinsed with running water, counterstained with hematoxylin for 5 min, differentiated with 1% hydrochloric acid alcohol for 5 s, and sealed with neutral resins. All sections were analyzed under a microscope (Olympus), and five non-overlapping positive regions were detected and analyzed using Image-Pro Plus 6.0.

### RT-qPCR

The total RNA was extracted from tissues and cells using the TRIzol reagent (Invitrogen, Carlsbad, CA, USA) and received treatment with gDNA Eraser at 4 ℃ for 2 h to aid the removal of genomic DNA. The reverse transcription of 1 μg total RNA was processed using PrimeScript RT Reagent kit (TaKaRa, Dalian, Liaoning, China) for the synthesis of the complementary DNA (cDNA). The 20 μL cDNA complex was prepared using SYBR Premix Ex Taq (TaKaRa). RT-qPCR was performed in Applied Biosystems 7500 Real-Time PCR System (Life Technologies). PCR primers are listed in Table 1. The relative gene expression was calculated using the 2^−ΔΔCt^ method [32], with GAPDH serving as the internal reference.

**Table 1:** PCR primer sequences

Gene	Sequence (5′–3′)
SNHG12	F: CCTTCTCTCGCTTCGGACTG
	R: TGCCAATAGCTGGTGTGCTT
PD-L1	F: CATTTGCTGAACGCCCCATA
	R: GTCCAGATGACTTCGGCCTT
USP8	F: CGACGCCAGAATGAAGAGGT
	R: TGGGCAGCAGGTTTAGAAGG
GAPDH	F: ATGGTTTACATGTTCCAATATGA
	R: TTACTCCTTGGAGGCCATGTGG

### Western blotting

Lysis solutions of tissues and cells were prepared using RIPA buffer, and the protein concentration was determined using the bicinchoninic acid reagent (Beyotime). Next, the total protein was loaded into 10% SDS-PAGE, left for 120 min, and transferred to polyvinylidene fluoride membranes (Sigma, St. Louis, MO, USA). After being blocked with 5% nonfat milk for 2 h and washed with Tris buffer containing Tween-20, membranes were incubated with the primary anti-PD-L1 (ab213524, 1:1000), anti-HuR (ab200342, 1:1000), anti-USP8 (ab228572, 1:1000), and anti-β-actin (ab8227, 1:1000) at 4 °C overnight. The following day, membranes were incubated with the secondary antibody (ab205718, 1:2000) at room temperature for 2 h. Proteins were visualized using an enhanced chemiluminescence assay. The gray value was analyzed using NIH Image J software (National Institutes of Health, Bethesda, MA, USA). All antibodies were provided by Abcam.

### Statistical analysis

All data were processed using SPSS21.0 software (IBM Corp, Armonk, NY, USA) and GraphPad Prism 8.0 software (GraphPad Software Inc., San Diego, CA, USA) for statistical analysis and graphing. Data conformed to normal distribution and homogeneity of variance. The *t*-test was adopted for pairwise comparisons of measurement data, one-way or two-way analysis of variance (ANOVA) was adopted for multigroup comparisons, and Tukey’s multiple comparison test was adopted for the post-test. Enumeration data were represented as case numbers. Fisher precision test was adopted for pairwise comparisons of enumeration data. The association between SNHG12 and the prognosis of patients with NSCLC was analyzed using Kaplan–Meier survivorship curve and log-rank test. The correlation between SNHG12 and PD-L1/USP8 was analyzed using Pearson correlation analysis. The *P* value was analyzed by two-sided test. A value of *P* < 0.05 was considered statistically significant, and a value of *P* < 0.01 indicated a difference with remarkable statistical significance.

## Results

### lncRNA SNHG12 was prominently expressed in NSCLC tissues and cells and was associated with the prognosis and clinicopathologic features of NSCLC patients

**Fig. 1:**
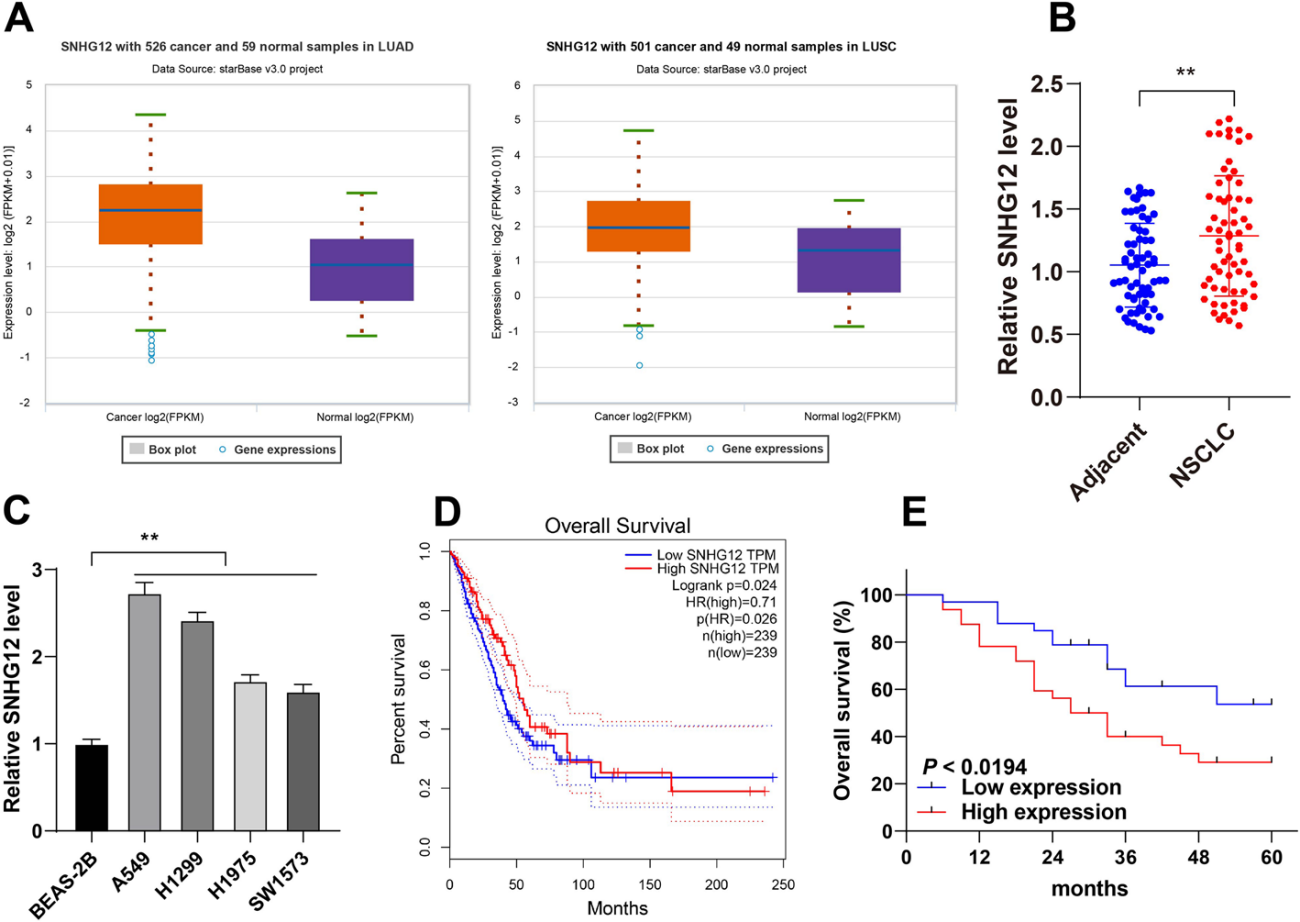
lncRNA SNHG12 was highly expressed in NSCLC tissues and cells and was associated with the prognosis and clinicopathologic features of patients with NSCLC. **A** lncRNA SNHG12 expression in NSCLC was predicted via the starBase database; **B** lncRNA SNHG12 expression in 65 pairs of NSCLC tissues and adjacent normal tissues was measured via RT-qPCR; **C** lncRNA SNHG12 expression in bronchial epithelial cells and NSCLC cell lines was measured via RT-qPCR; **D** association between lncRNA SNHG12 expression and prognosis of patients with NSCLC was predicted via the GEPIA database; **E** association between lncRNA SNHG12 expression and the prognosis of 65 patients with NSCLC was analyzed via Kaplan–Meier survivorship curve. *N* = 65, experiments were performed three times, **P < 0.01. Data in **C** are represented as mean ± standard deviation. Pairwise comparisons in **B** were analyzed using paired *t*-test and in **E** were analyzed using log-rank test, and multigroup comparisons in **C** were analyzed using Tukey’s multiple comparison test

Accumulating evidence shows that lncRNAs play a role in the initiation and development of NSCLC [33], and lncRNA SNHG12 is overexpressed in NSCLC and promotes NSCLC progression [14, 34]. However, the role of lncRNA SNHG12 in NSCLC was yet to be determined. Through the starBase database, a strong expression of lncRNA SNHG12 was predicted in NSCLC (Fig. 1A). Next, lncRNA SNHG12 expression was measured in NSCLC tissues and cells, the findings of which revealed increased expression of lncRNA SNHG12 in NSCLC tissues and cells (*P* < 0.01, Fig. 1B, C). In addition, the GEPIA database predicted that the high expression level of SNHG12 is correlated with poor prognosis (Fig. 1D). Then, 65 patients were grouped into the high lncRNA SNHG12 and low lncRNA SNHG12 groups, with the mean lncRNA SNHG12 expression serving as the critical threshold to confirm the association between lncRNA SNHG12 and the prognosis and clinicopathologic features of patients with NSCLC. The results showed that there exists a link between lncRNA SNHG12 expression and tumor size, histological grade, lymph node metastasis, and tumor–node–metastasis stage (*P* < 0.05, Table 2). Kaplan–Meier survivorship analysis revealed that higher expression of lncRNA SNHG12 was associated with shorter overall survival of patients with NSCLC (*P* < 0.05, Fig. 1E). These results suggest that there is a remarkable expression of lncRNA SNHG12 in NSCLC tissues and cells and that this expression is related to the prognosis and clinicopathologic features of patients with NSCLC.

**Table 2:** Correlation between lncRNA SNHG12 and clinicopathological characteristics of patients with NSCLC

Characteristic	Number	lncRNA SNHG12		*P* value
	*N* = 65	Low expression (*N* = 33)	High expression (*N* = 32)	
Age (years)				0.540
< 55	30	14	16	
≥ 55	35	19	16	
Sex				0.531
Male	31	17	14	
Female	34	16	18	
Histological subtype				0.524
Squamous cell carcinoma	29	16	13	
Adenocarcinoma	36	17	19	
Tumor size				0.015
< 3 cm	26	18	8	
≥ 3 cm	39	15	24	
Smoking history				0.384
Ever	33	15	18	
Never	32	18	14	
Histological grade				0.017
Well and moderately	28	19	9	
Poorly	37	14	23	
Lymph node metastasis				0.018
Negative	30	20	10	
Positive	35	13	22	
TNM stage				0.031
I–II	27	18	9	
III–IV	38	15	23	

The chi-square test was used for comparisons among data. A value of *P* < 0.05 indicates statistical significance

### lncRNA SNHG12 downregulation repressed proliferation and promoted apoptosis of NSCLC cells

**Fig. 2:**
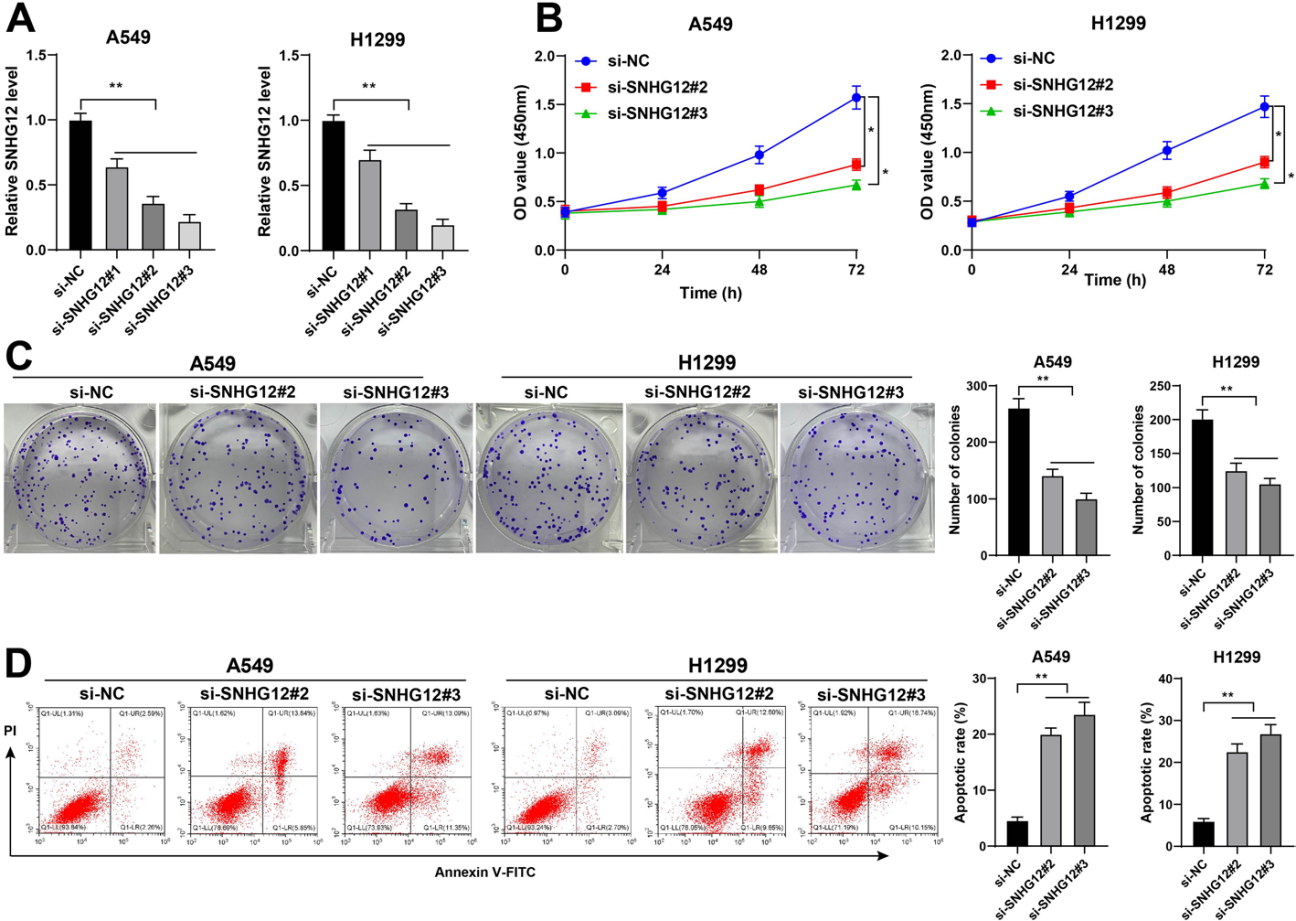
lncRNA SNHG12 downregulation repressed proliferation and promoted apoptosis of NSCLC cells. A549 or H1299 cells were transfected with SNHG12 siRNA (si-SNHG12), with NC siRNA (NC siRNA) as the negative control. **A** lncRNA SNHG12 expression in NSCLC cells was measured via RT-qPCR; **B-C** cell proliferation was assessed via CCK-8 and colony-formation assays; **D** apoptosis was assessed via flow cytometry. Cell experiments were performed three times, *P < 0.05, **P < 0.01. Data are represented as mean ± standard deviation. Multigroup comparisons in **A**, **C**, and **D** were analyzed using one-way ANOVA and in **B** were analyzed using two-way ANOVA, followed by Tukey’s multiple comparison test

To confirm whether lncRNA SNHG12 affects NSCLC development, we designed three strands of siRNAs (si-SNHG12#1, si-SNHG12#2, and si-SNHG12#3) and transfected them into A549 and H1299 cells with comparatively high expression of lncRNA SNHG12 with aims of downregulating lncRNA SNHG12 expression in cells (*P* < 0.01, Fig. 2A). Next, we selected si-SNHG12#2 and si-SNHG12#3 for subsequent experiments, owing to their comparatively higher knockdown efficiency. The results showed a significant decline in cell proliferation upon silencing of lncRNA SNHG12 (*P* < 0.05, Fig. 2B, C) while the apoptosis rate was markedly increased (*P* < 0.01, Fig. 2D). These results suggested that lncRNA SNHG12 downregulation suppressed proliferation and promoted apoptosis of NSCLC cells.

### lncRNA SNHG12 downregulation blocked the immune escape of NSCLC

**Fig. 3:**
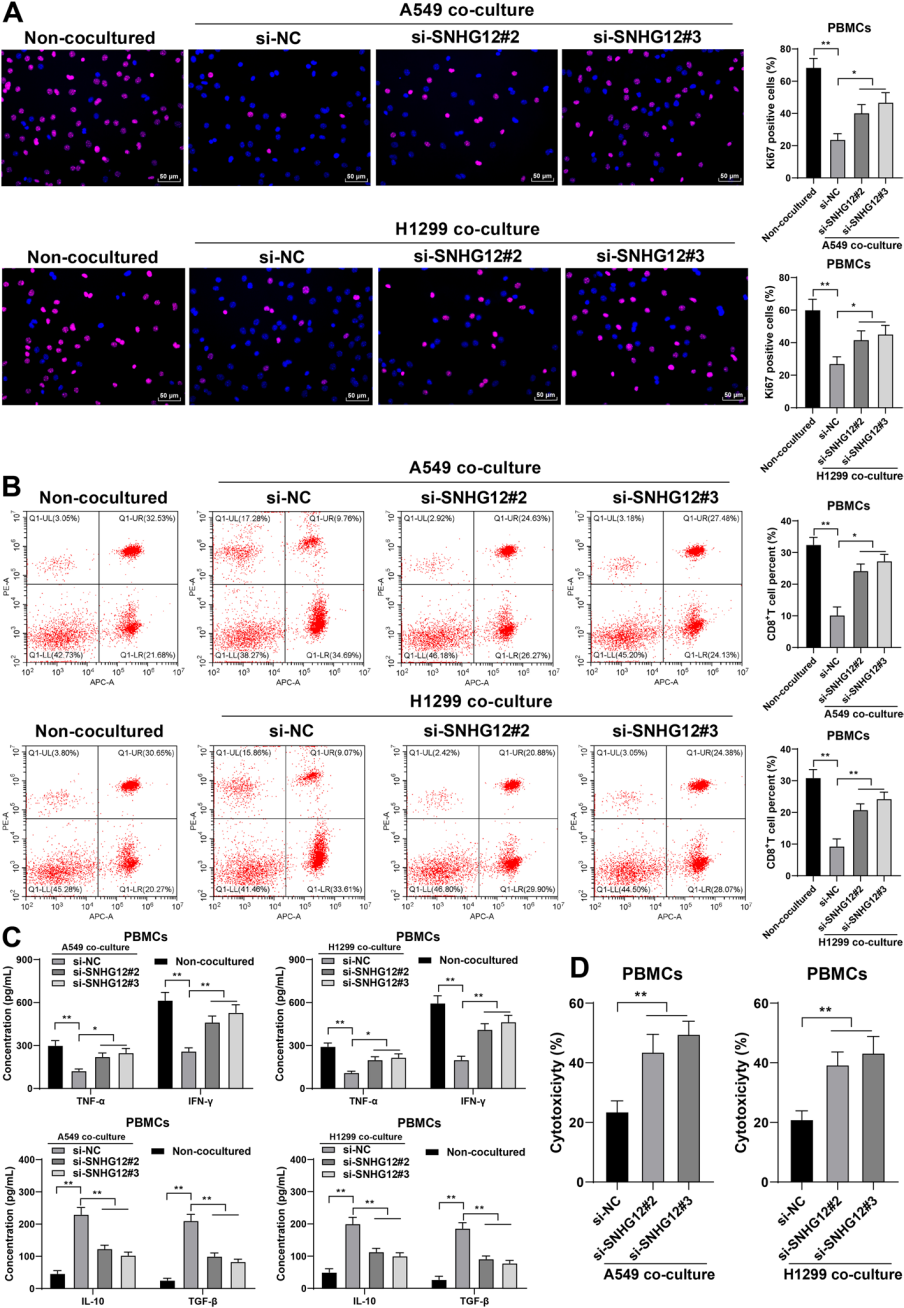
lncRNA SNHG12 downregulation blocked the immune escape of NSCLC. A549 or H1299 cells in different treatment groups were co-cultured with isolated PBMCs. **A** PBMC proliferation was observed via Ki67 staining; **B** percentage of CD8^+^ T cells in PBMCs was determined via flow cytometry; **C** concentrations of TNF-α, IFN-γ, IL-10, and TGF-β were determined via ELISA; **D** cytotoxicity of PBMCs was detected via LDH cytotoxicity assay. Cell experiments were performed in triplicates, *P < 0.05, **P < 0.01. Data were represented as mean ± standard deviation. Multigroup comparisons in **A**, **B**, and **D** were analyzed using one-way ANOVA and in **C** were analyzed using two-way ANOVA, followed by Tukey’s multiple comparison test

It has been established that immune escape is evidenced to fuel cancer initiation and development [35]. To confirm the role of lncRNA SNHG12 in the immune escape of NSCLC, we co-cultured PBMCs with A549 or H1299 cells. The results showed that PBMC proliferation was inhibited in the co-culture system (*P* < 0.01, Fig. 3A), the percentage of CD8^+^ T cells was decreased (*P* < 0.01, Fig. 3B), TNF-α and IFN-γ levels in the supernatant of PBMCs were significantly reduced, and IL-10 and TGF-β levels in the supernatant of PBMCs were significantly increased (*P* < 0.01, Fig. 3C). Silencing lncRNA SNHG12 resulted in an increase in PBMC proliferation and the percentage of CD8^+^ T cells (*P* < 0.05, Fig. 3A, B), along with increased TNF-α and IFN-γ levels, decreased IL-10 and TGF-β levels (*P* < 0.05, Fig. 3C), and augmented cytotoxicity of PBMCs (*P* < 0.05, Fig. 3D). These results suggest that lncRNA SNHG12 downregulation blocked the immune escape of NSCLC.

### lncRNA SNHG12 bound to HuR to increase mRNA stability of PD-L1

**Fig. 4:**
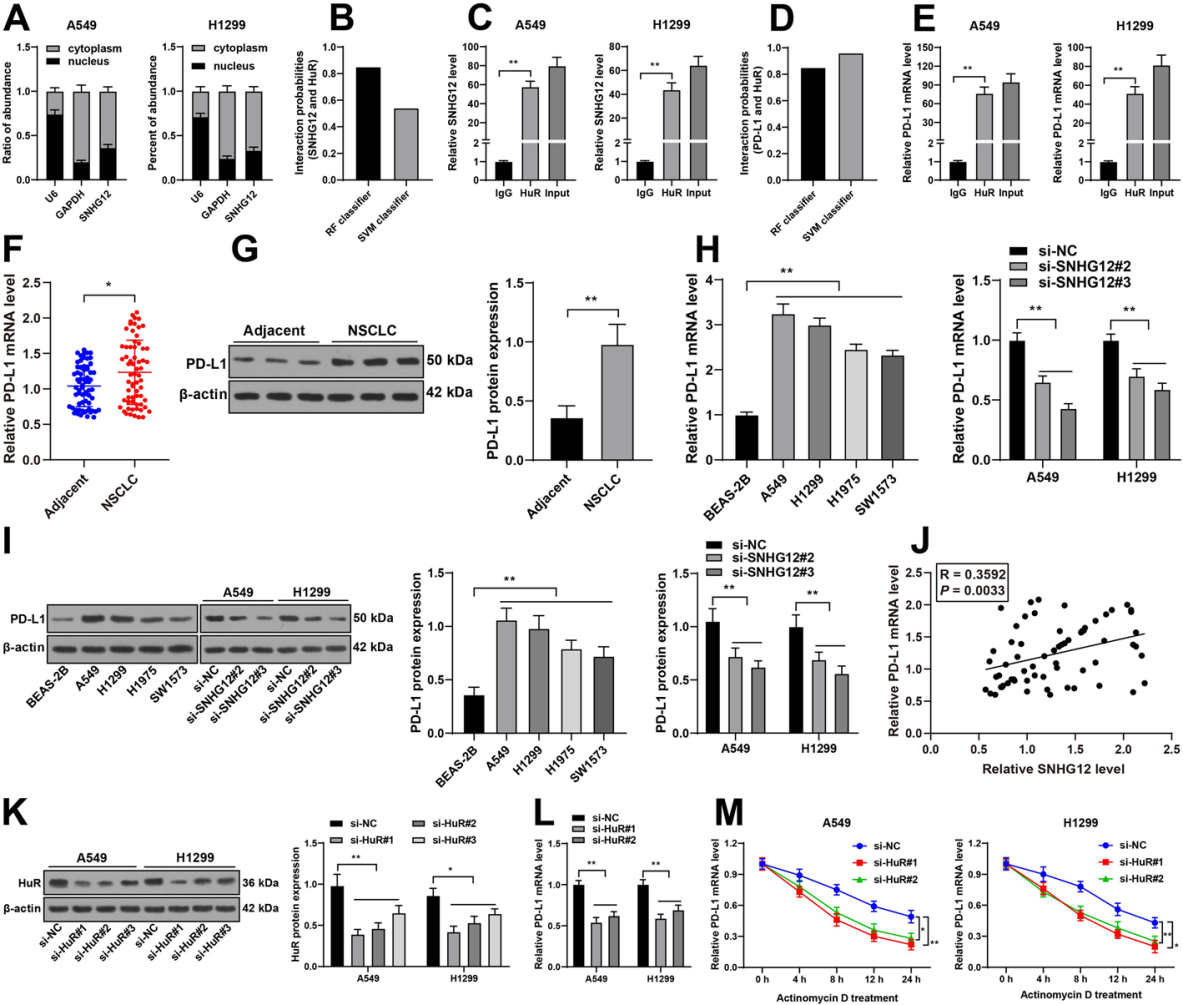
lncRNA SNHG12 bound to HuR to increase mRNA stability of PD-L1. **A** Localization of lncRNA SNHG12 in NSCLC cells was analyzed via the subcellular fractionation assay; **B** probability of lncRNA SNHG12 binding to HuR was predicted via the RNA–Protein Interaction Prediction (RPISeq) database; **C** the binding relationship between SNHG12 and HuR was analyzed using RIP assay; **D** probability of HuR binding to PD-L1 was predicted via the RPISeq database; **E** the binding relationship between PD-L1 and HuR was analyzed using RIP assay; **F**–**I** PD-L1 expression in NSCLC tissues and cells was measured via RT-qPCR and western blotting; **J** correlation between lncRNA SNHG12 and PD-L1 was analyzed via Pearson correlation analysis; A549 or H1299 cells were transfected by three strands of HuR siRNA (si-HuR), with si-NC as the negative control; **K** protein expression of HuR was detected via western blotting; **L**, **M** expression and half-life period of PD-L1 mRNA were determined via RT-qPCR. *N* = 65, cell experiments were performed three times, *P < 0.05, **P < 0.01. Data in **A**–**E**, **G**–**I**, and **K**–**M** are represented as mean ± standard deviation. Pairwise comparisons in **F** and **G** were analyzed using *t*-test; multigroup comparisons in **C**, **E**, **H**, and **I** were analyzed using one-way ANOVA, and in **H**, **I**, and **K**–**M** were analyzed using two-way ANOVA, followed by Tukey’s multiple comparison test

Next, the downstream mechanism of lncRNA SNHG12 was explored. The subcellular fractionation assay identified the cytoplasmic localization of lncRNA SNHG12 (Fig. 4A). Prior studies suggested that lncRNAs in the cytoplasm can bind to cytoplasmic RNAs to influence the expression patterns of downstream target genes [36, 37]. As indicated by existing literature, lncRNA SNHG12 potently binds to HuR [18]. The database prediction indicated a comparatively high probability of lncRNA SNHG12 binding to HuR (the RF classifier and SVM classifier scores) (Fig. 4B). RIP assay validated the binding relationship that exists between lncRNA SNHG12 and HuR (*P* < 0.01, Fig. 4C). HuR is capable of enhancing the mRNA stability of PD-L1 [21]. Moreover, PD-L1/PD-1 is a crucial part of cancer immune escape [20]. The database prediction indicated a comparatively high probability of HuR binding to PD-L1 mRNA (Fig. 4D), and the RIP assay also revealed that HuR could bind to PD-L1 mRNA (*P* < 0.01, Fig. 4E). Subsequently, findings from RT-qPCR and western blot revealed a prominent expression of PD-L1 in NSCLC tissues and cells and its decay secondary to lncRNA SNHG12 silencing (*P* < 0.05, Fig. 4F–I). In addition, there was an evident positive association between lncRNA SNHG12 expression and PD-L1 expression (*P* < 0.05, Fig. 4J). To confirm the promotive effect of lncRNA SNHG12 on PD-L1 expression via binding to HuR, HuR siRNA (si-HuR) was transfected into NSCLC cells to downregulate HuR expression (*P* < 0.05, Fig. 4K). HuR knockdown led to a decrease in the expression and half-life period of PD-L1 mRNA (*P* < 0.05, Fig. 4L, M). Collectively, these results suggested that the binding of lncRNA SNHG12 to HuR enhances the mRNA stability of PD-L1.

### lncRNA SNHG12 bound to HuR to increase mRNA stability and expression of USP8

**Fig. 5:**
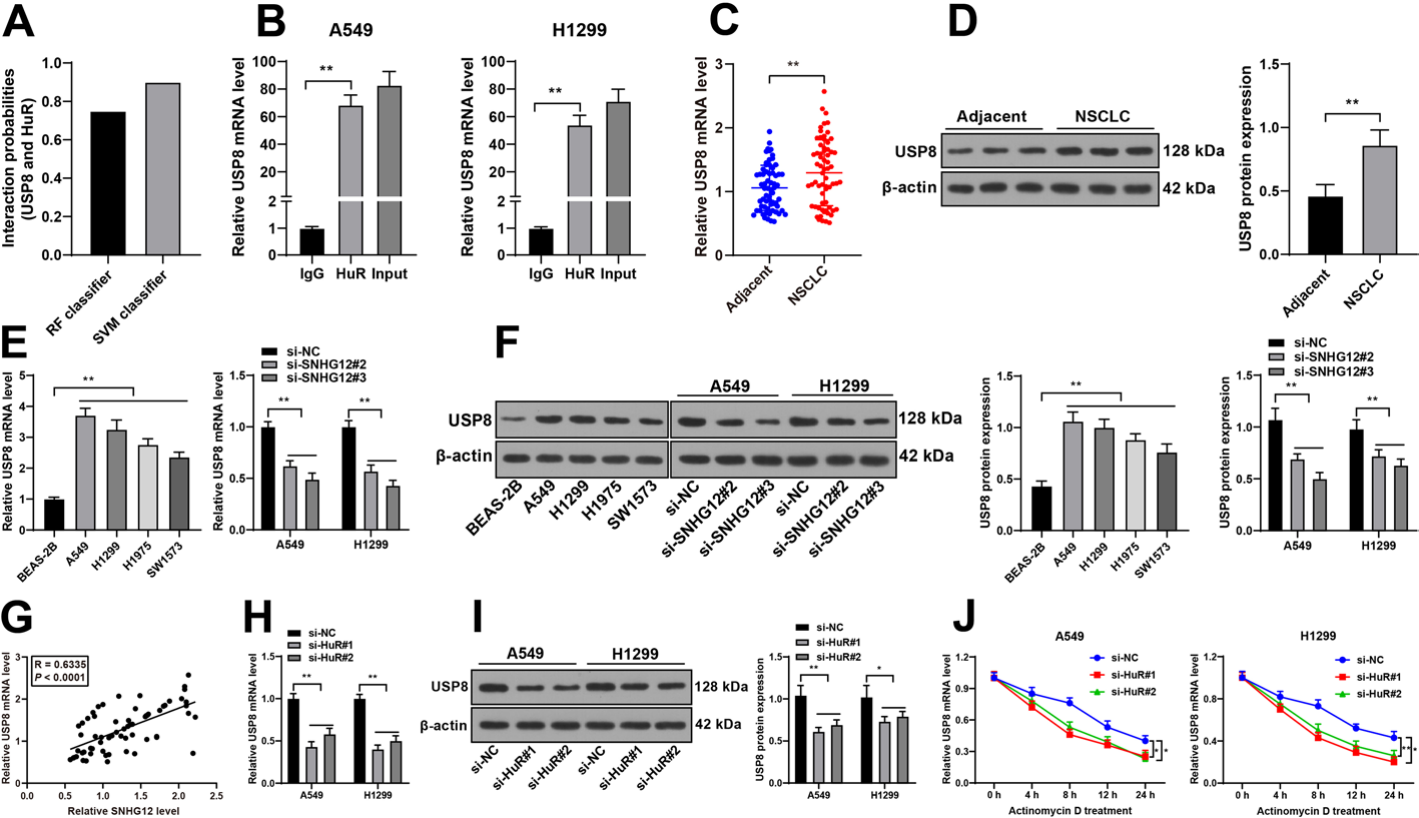
lncRNA SNHG12 bound to HuR to increase mRNA stability and expression of USP8. **A** Probability of HuR binding to USP8 was predicted via the RPISeq database; **B** the binding relationship between USP8 and HuR was analyzed using RIP assay; **C**–**F** USP8 expression in NSCLC tissues and cells was measured via RT-qPCR and western blotting; **G** correlation between lncRNA SNHG12 and USP8 was analyzed via Pearson correlation analysis; **H**, **I** USP8 expression in NSCLC cells was measured via RT-qPCR and western blotting; **J** half-life period of USP8 mRNA in NSCLC cells was assessed via RT-qPCR. *N* = 65, cell experiments were performed three times, *P < 0.05, **P < 0.01. Data in **A**, **B**, **D**–**F**, and **H**–**J** are represented as mean ± standard deviation. Pairwise comparisons in **C** and **D** were analyzed using *t*-test; multigroup comparisons in **B**, **E**, and **F** were analyzed using one-way ANOVA, and in **E**, **F**, and **H**–**J** were analyzed using two-way ANOVA, followed by Tukey’s multiple comparison test

In addition, increased HuR collaborates with USP8 mRNA, resulting in an increase in mRNA stability and protein expression of USP8 [22]. USP8 downregulation leads to suppression in proliferation, migration, and invasion while promoting apoptosis of LC cells [23, 24]. The database prediction indicated a comparatively high probability of HuR binding to USP8 (Fig. 5A). RIP assay further verified that HuR could bind to USP8 mRNA in NSCLC cells (*P* < 0.01, Fig. 5B). Following RT-qPCR and western blot, the results were indicative of upregulated USP8 levels in NSCLC tissues and cells and USP8 expression was decreased as a result of lncRNA SNHG12 downregulation (*P* < 0.01, Fig. 5C–F). In addition, lncRNA SNHG12 expression was positively correlated with USP8 expression (*P* < 0.01, Fig. 5G). Subsequently, a change in USP8 expression was detected in the si-HuR group; USP8 expression and half-life period of USP8 mRNA were both reduced upon HuR downregulation (*P* < 0.05, Fig. 5H–J). These results suggest that lncRNA SNHG12 binds to HuR, resulting in an increase in mRNA stability and expression of USP8.

### USP8 stabilized the protein level of PD-L1 through deubiquitination

**Fig. 6:**
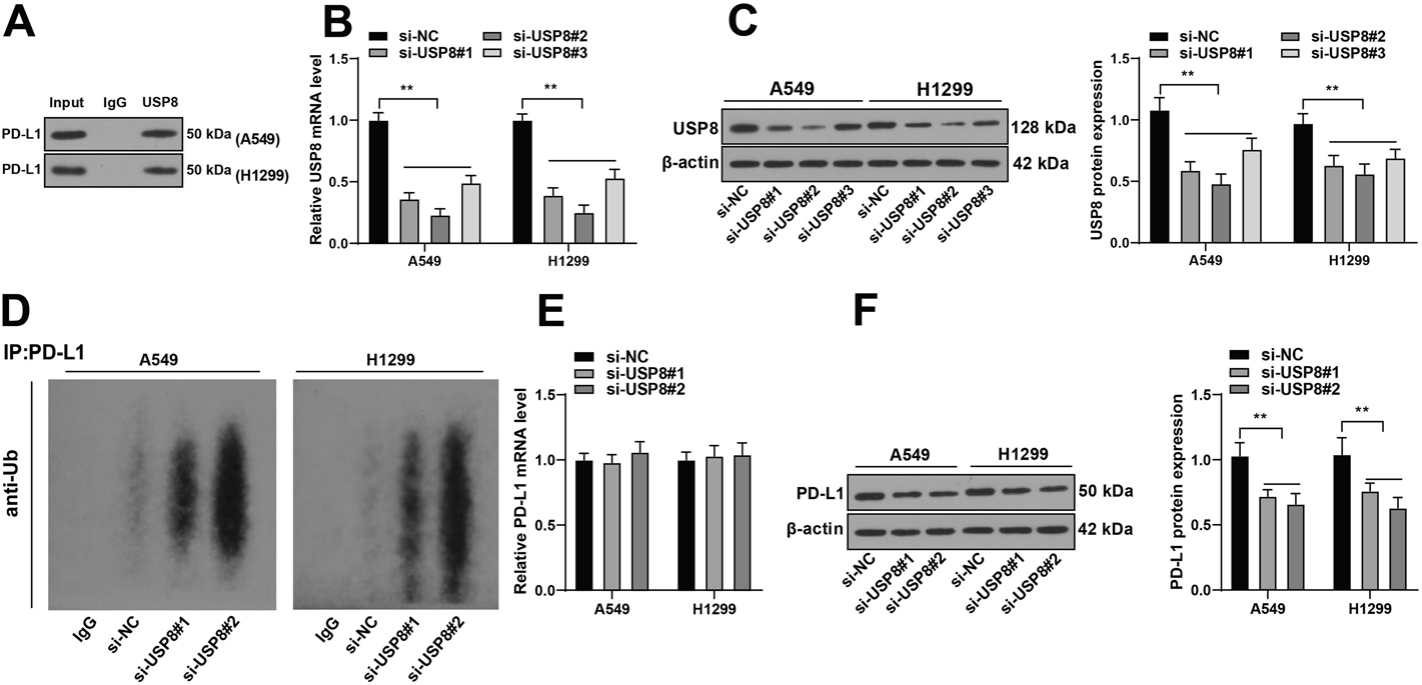
USP8 stabilized the protein level of PD-L1 through deubiquitination. **A** The binding between PD-L1 and USP8 was analyzed via Co-IP assay; three strands of USP8 siRNA (si-USP8) were transfected into A549 or H1299 cells, with si-NC as the negative control; **B**, **C** USP8 expression in NSCLC cells was detected via RT-qPCR and western blotting; **D** ubiquitination level of PD-L1 in NSCLC cells was detected via Co-IP and western blotting; **E**, **F** PD-L1 expression in NSCLC cells was detected RT-qPCR and western blotting. Cell experiments were performed three times, **P < 0.01. Data are represented as mean ± standard deviation. Multigroup comparisons in **B**, **C**, **E**, and **F** were analyzed using two-way ANOVA, followed by Tukey’s multiple comparison test

USP8 is a crucial deubiquitinase that participates in protein degradation [38]. Deubiquitinase regulates the protein stability of PD-L1 to affect the immune escape of tumors [39, 40]. We inferred that USP8 could potentially regulate the protein level of PD-L1 through deubiquitination. First, Co-IP assay was performed, the findings of which confirmed an interaction between USP8 and PD-L1 (Fig. 6A). Next, transfected USP8 siRNA (si-USP8) was transfected into NSCLC cells to downregulate USP8 expression (*P* < 0.01, Fig. 6B, C). si-USP8#1 and si-USP8#2 were found to have higher silencing efficiency and, as a result, were selected for subsequent experiments. USP8 downregulation led to an increase in ubiquitination level of PD-L1 (*P* < 0.01, Fig. 6D), no significant change in PD-L1 mRNA level (Fig. 6E), and decreased protein level of PD-L1 (*P* < 0.01, Fig. 6F). These results suggest that USP8 stabilized the protein level of PD-L1 at the post-transcriptional level through deubiquitination.

### USP8 overexpression attenuated the inhibition of silencing lncRNA SNHG12 on the immune escape of NSCLC

**Fig. 7:**
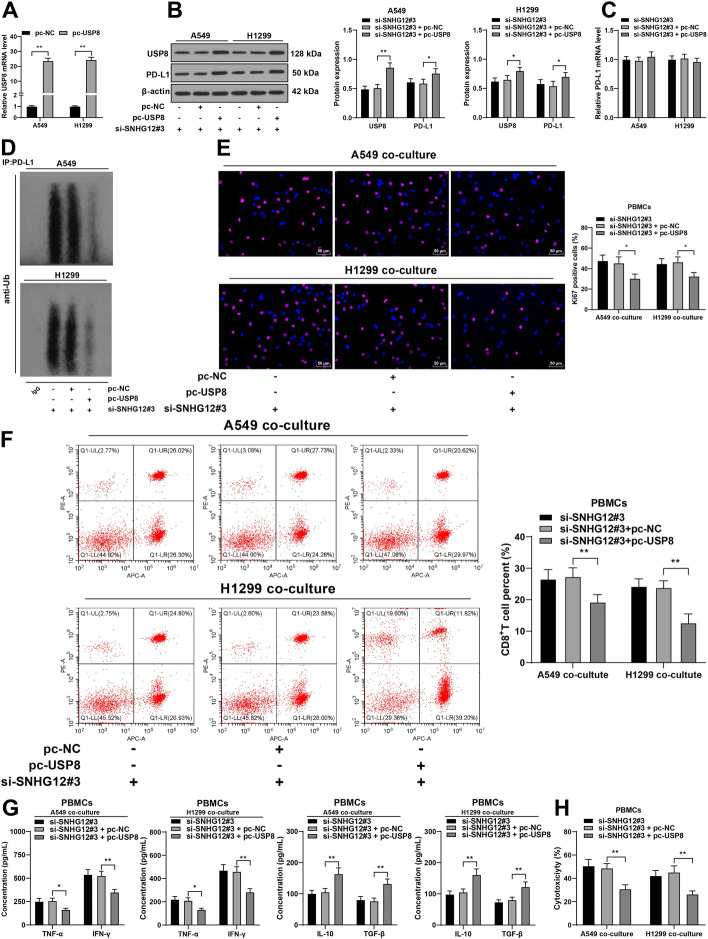
USP8 overexpression attenuated the inhibition of silencing lncRNA SNHG12 on the immune escape of NSCLC. A549 or H1299 cells were transfected with USP8 pcDNA3.1 (pc-USP8), with NC pcDNA3.1 (pc-NC) as the negative control. **A** mRNA level of USP8 in NSCLC cells was measured via RT-qPCR; **B** protein expression of USP8 and PD-L1 was detected via western blotting; **C** mRNA level of PD-L1 was measured via RT-qPCR; **D** ubiquitination level of PD-L1 in NSCLCs was determined via western blotting; A549 or H1299 cells in different treatment groups were co-cultured with isolated PBMCs. **E** PBMC proliferation was observed via Ki67 staining; **F** percentage of CD8^+^ T cells in PBMCs was determined via flow cytometry; **G** concentrations of TNF-α, IFN-γ, IL-10, and TGF-β were determined via ELISA; **H** cytotoxicity of PBMCs was detected via LDH cytotoxicity assay. Cell experiments were performed three times, *P < 0.05, **P < 0.01. Data are represented as mean ± standard deviation. Multigroup comparisons in **A**–**C** and **E**–**H** were analyzed using two-way ANOVA, followed by Tukey’s multiple comparison test

Thereafter, the role of USP8 in lncRNA SNHG12-regulated immune escape of NSCLC was determined. We transfected USP8 pcDNA3.1 (pc-USP8) into NSCLC cells to upregulate USP8 expression (*P* < 0.05, Fig. 7A, B), followed by combined treatment with si-SNHG12#3 in NSCLC cells. The findings showed that USP8 overexpression resulted in no change in PD-L1 mRNA level, decreased ubiquitination level of PD-L1, and increased protein level of PD-L1 (*P* < 0.05, Fig. 7B–D). Next, NSCLC cells were co-cultured with PBMCs. It was found that USP8 overexpression led to the inhibition of PBMC and CD8^+^ T-cell proliferation (*P* < 0.05, Fig. 7E, F), a decline in TNF-α and IFN-γ levels, an increase in IL-10 and TGF-β levels (*P* < 0.05, Fig. 7G), and weakened cytotoxicity of PBMCs (*P* < 0.01, Fig. 7H). These results suggest that USP8 overexpression attenuated the inhibition of silencing lncRNA SNHG12 on the immune escape of NSCLC.

### PD-L1 overexpression attenuated the inhibition of silencing lncRNA SNHG12 on the immune escape of NSCLC

**Fig. 8:**
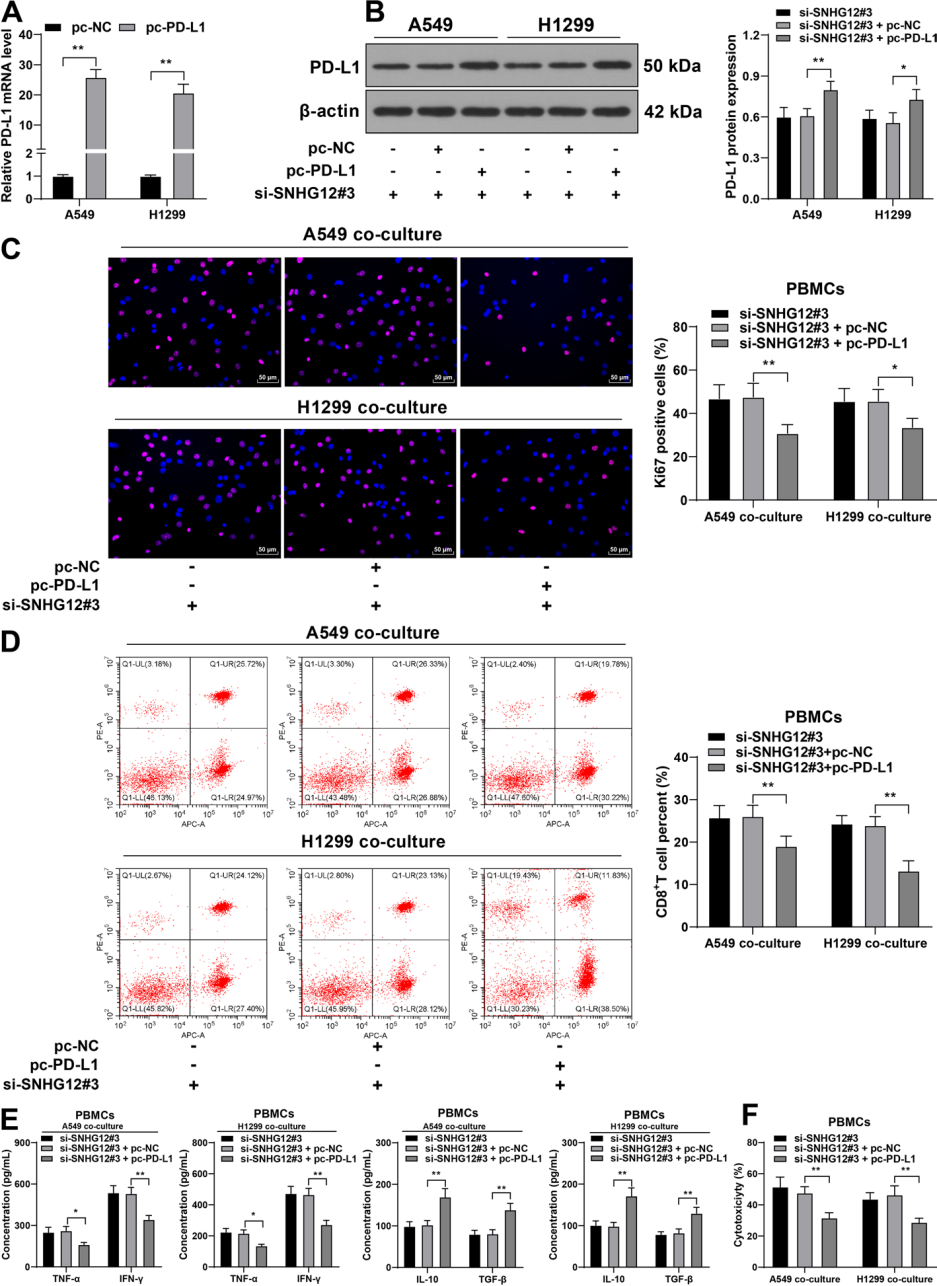
PD-L1 overexpression attenuated the inhibition of silencing lncRNA SNHG12 on immune escape of NSCLC. A549 or H1299 cells were transfected with PD-L1 pcDNA3.1 (pc-PD-L1), with NC pcDNA3.1 (pc-NC) as the negative control. **A**, **B** PD-L1 expression in NSCLCs was measured via RT-qPCR and western blotting; A549 or H1299 cells in different treatment groups were co-cultured with isolated PBMCs. **C** PBMC proliferation was observed via Ki67 staining; **D** percentage of CD8^+^ T cells in PBMCs was detected via flow cytometry; **E** concentrations of TNF-α, IFN-γ, IL-10, and TGF-β were determined via ELISA; **F** cytotoxicity of PBMCs was detected via LDH cytotoxicity assay. Cell experiments were performed three times, *P < 0.05, **P < 0.01. Data are represented as mean ± standard deviation. Multigroup comparisons in **A**–**F** were analyzed using two-way ANOVA, followed by Tukey’s multiple comparison test

Next, the role of PD-L1 in lncRNA SNHG12-regulated immune escape of NSCLC was explored. PD-L1 pcDNA3.1 (pc-PD-L1) was transfected into NSCLC cells to upregulate PD-L1 expression (*P* < 0.05, Fig. 8A, B), after which it was combined with si-SNHG12#3 to treat NSCLC cells. NSCLC cells that received treatment were then co-cultured with PBMCs. On the basis of the results, PD-L1 overexpression led to inhibited PBMC and CD8^+^ T-cell proliferation (*P* < 0.05, Fig. 8C, D), downregulated TNF-α and IFN-γ levels, increased IL-10 and TGF-β levels (*P* < 0.05, Fig. 8E), and weakened cytotoxicity of PBMCs (*P* < 0.01, Fig. 8F). These results suggest that PD-L1 overexpression attenuated the inhibition of silencing lncRNA SNHG12 on the immune escape of NSCLC.

### lncRNA SNHG12 increased expression stability of PD-L1 via HuR to facilitate NSCLC cell growth and immune escape in vivo

**Fig. 9:**
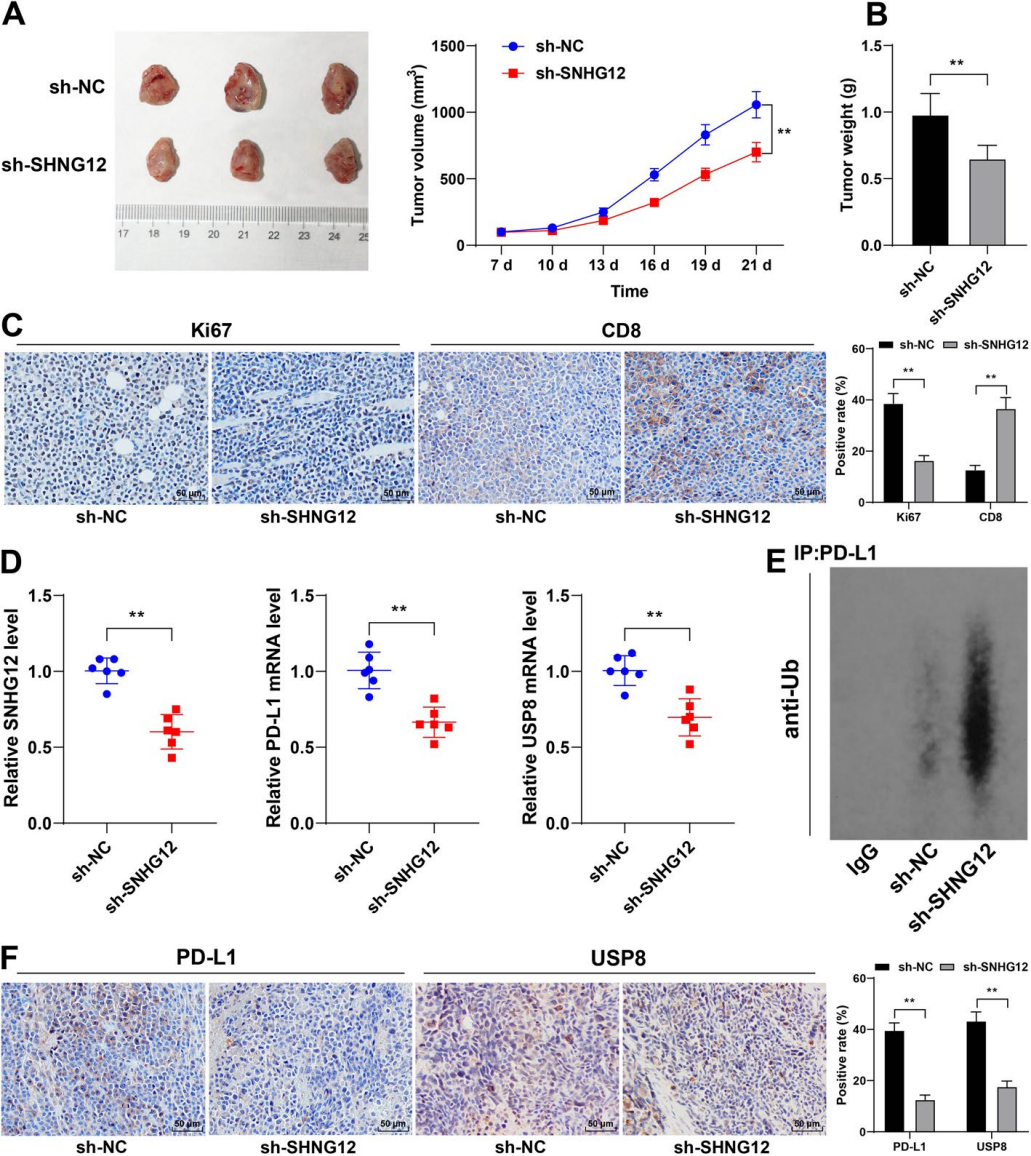
lncRNA SNHG12 increased expression stability of PD-L1 via HuR to facilitate NSCLC cell growth and immune escape in vivo. **A**, **B** Volume and weight of representative xenograft tumors; **C** positive rates of Ki67 and CD8 were determined via immunohistochemistry; **D** SNHG12, PD-L1, and USP8 expression was measured via western blotting; **D** SNHG12, PD-L1, and USP8 levels were determined via RT-qPCR; **E** ubiquitination level of PD-L1 in xenograft tumors was determined via Western blot analysis; **F** positive rates of PD-L1 and USP8 were determined via immunochemistry. **P < 0.01. Data are represented as mean ± standard deviation. Pairwise comparisons in **B** and **D** were analyzed using * t*-test, and multi-group comparisons in **A**, **C**, and **F** were analyzed using two-way ANOVA, followed by Tukey’s multiple comparison test

Finally, we validated the role of lncRNA SNHG12 in the immune escape of NSCLC in vivo. We injected A549 cells with stable low expression of lncRNA SNHG12 into C57BL/6 mice. The results found that lncRNA SNHG12 downregulation led to reduced tumor volume and weight (*P* < 0.01, Fig. 9A, B) and Ki67 positive rate, and increased CD8 positive rate (*P* < 0.01, Fig. 9C), along with reduced levels of SNHG12, PD-L1, and USP8 (*P* < 0.01, Fig. 9D), elevated ubiquitination level of PD-L1 (*P* < 0.01, Fig. 9E), and decreased   positive rate PD-L1 and USP8 (*P* < 0.01, Fig. 9F). These results suggest that lncRNA SNHG12 increased the expression stability of PD-L1 via HuR to facilitate NSCLC cell growth and immune escape in vivo.

## Discussion

**Fig. 10:**
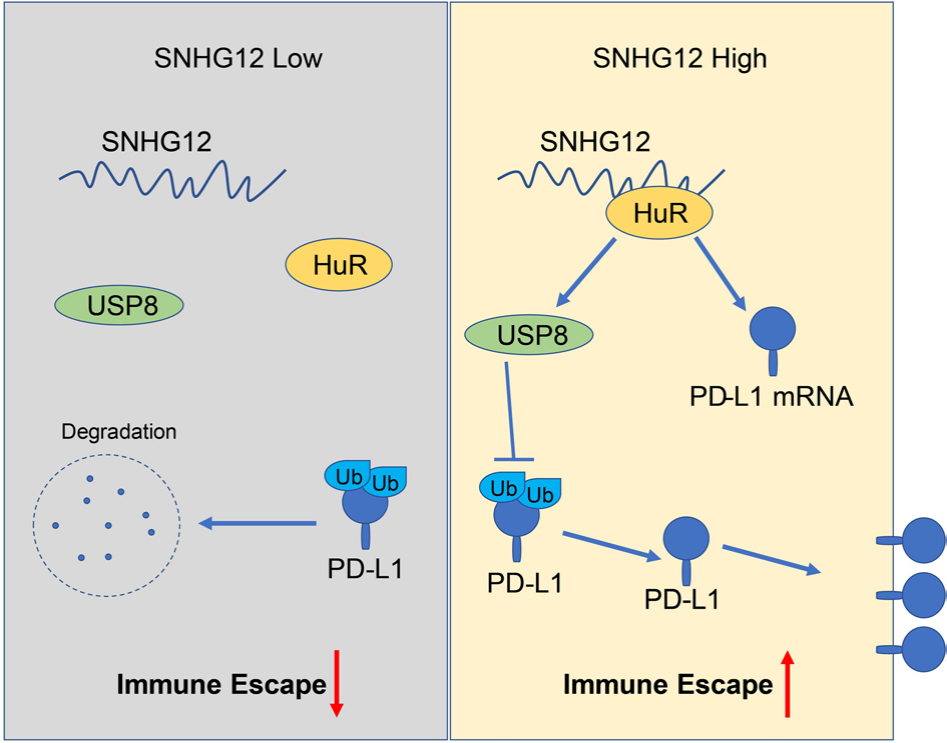
Mechanism of lncRNA SNHG12 in immune escape of NSCLC. SNHG12 was highly expressed in NSCLC. On the one hand, SNHG12 bound to HuR to improve mRNA stability and expression of PD-L1. On the other hand, lncRNA SNHG12 bound to HuR to improve mRNA stability and expression of USP8. USP8-mediated deubiquitination increased the protein level of PD-L1, consequently promoting immune escape of NSCLC

Immunotherapy centered on the host immune response has promising prospects in finding NSCLC treatment [4]. Unfortunately, the tumor microenvironment of NSCLC enables tumors to escape from immune attacks during immunotherapy [5, 41]. lncRNAs have been shown to play a fundamental role in cancer immunomodulation and could serve as immunotherapy targets [42]. Our findings from the present study revealed that lncRNA SNHG12 improves mRNA stability and expression of PD-L1 and USP8, and USP8-mediated deubiquitination increases the protein level of PD-L1, resulting in the subsequent enhancement of immune escape in NSCLC (Fig. 10).

The overexpression of lncRNA SNHG12 in NSCLC tissues and cells and its ability to positively regulate NSCLC progression have been previously highlighted [13–15, 34]. Accordingly, the starBase database predicted the presence of increased levels of lncRNA SNHG12 in NSCLC tissues and cell lines. Next, the 65 patients with NSCLC were grouped into the high/low lncRNA SNHG12 expression groups, and lncRNA SNHG12 expression was determined in relation to histological grade, lymph node metastasis, and TNM stage. Kaplan–Meier survivorship analysis suggested that higher lncRNA SNHG12 expression was associated with shorter survival of patients with NSCLC. In accordance, it has been previously determined that lncRNA SNHG12 serves as a negative predictor of clinicopathological characteristics and survival in renal cell, prostate, and nasopharyngeal carcinomas [43–45]. Similarly, our results found that there is significant expression of lncRNA SNHG12 in NSCLC tissues and cells and that there is an association between this upregulated expression and the prognosis and clinicopathological characteristics of patients with NSCLC.

Subsequently, lncRNA SNHG12 expression was downregulated in A549 and H1299 cells using si-SNHG12 to determine its role in NSCLC cell functions. Our results suggested that silencing lncRNA SNHG12 suppressed proliferation and promoted apoptosis of NSCLC cells. As tumor-specific immunosuppressive cells, CD8^+^ T cells serve as a barrier protecting against the killing of cancer cells [46, 47]. Furthermore, PBMCs were co-cultured with si-SNHG12-treated A549 and H1299 cells, upon which silencing lncRNA SNHG12 was found to promote PBMC proliferation and enhance the ratio of CD8^+^ T cells, along with increases in TNF-α and IFN-γ levels and PBMC cytotoxicity, and decreases in IL-10 and TGF-β levels. Our in vivo experiments further suggested that tumor weight and volume were decreased secondary to the knockdown of lncRNA SNHG12, which also led to reduced positive rates of Ki67 and increased positive rates of CD8. A pioneering prior study has identified the functionality of lncRNA SNHG12 in the polarization of tumor-primed immune cells through the unfolded protein response, leading to immune escape of tumors [12]. For instance, lncRNA SNHG12 promotes immune escape of ovarian cancer by activating the crosstalk between M2 macrophage and ovarian cancer cells [48]. Collectively, our study was the first to demonstrate that the inhibition of lncRNA SNHG12 blocks immune escape of NSCLC, limiting tumor growth.

Subsequently, the downstream mechanism of lncRNA SNHG12 was explored in NSCLC. lncRNA SNHG12 is known as a cytoplasmic lncRNA that binds to cytoplasmic RNA‐binding proteins, further regulating downstream protein targets [16]. The subcellular fractionation assay confirmed the localization of lncRNA SNHG12 in the cytoplasm. It is worth noting that lncRNA SNHG12 can bind to HuR [17, 18]. HuR is prominently expressed in NSCLC to drive its progression, and HuR inhibitor-induced cytotoxicity increases NSCLC cell apoptosis [49, 50]. RIP assay revealed the binding relationship between lncRNA SNHG12 and HuR. Moreover, cytoplasmic localization of HuR allows itself to affect the stability and translation of cancer-related mRNAs [51]. PD-L1 is enriched in NSCLC, and high expression of PD-L1 signifies shorter survival of patients with NSCLC [52]. A prior study reported that HuR increases mRNA stability of PD-L1, resulting in an enhanced tumor immunosuppressive microenvironment in NSCLC [21]. The binding relationship between HuR and PD-L1 mRNA was established with the application of RIP assay. NSCLC tissues and cells were observed to have evidently increased PD-L1; however, this finding was reversed in response to silencing lncRNA SNHG12, and HuR knockdown reduced the mRNA level and half-life period of PD-L1. Meanwhile, HuR has been shown to upregulate mRNA stability and protein expression of USP8 in a prior study [22]. USP8 inhibitor has also displayed satisfactory antitumor efficacy in NSCLC [23, 24]. RIP assay provided evidence of the binding relationship between HuR and USP8 mRNA. NSCLC tissues and cells presented with elevated USP8, but this finding was reversed in response to silencing lncRNA SNHG12, and HuR knockdown reduced the mRNA level and half-life period of USP8. Generally, our results demonstrated that lncRNA SNHG12 increased mRNA stability and expression of PD-L1 and USP8 by binding to HuR.

As a deubiquitinase, USP8 improves the stability of target oncoproteins by preventing ubiquitin-dependent degradation [25, 26]. The binding of PD-L1 to its receptor PD-1 counteracts T-cell-activating signals, resulting in the suppression of antitumor immune responses [53, 54]. Deubiquitination impairs protein expression of PD-L1, which leads to enhanced immune responses in tumors [39, 40]. Accordingly, silencing USP8 increased ubiquitination level and decreased protein level of PD-L1, without significantly affecting PD-L1 mRNA level. Subsequently, USP8 or PD-L1 were in NSCLC cells, with the transfected cells subsequently co-cultured with PBMCs. Our results suggested that overexpression of USP8 or PD-L1 led to the inhibition of PBMC proliferation and reduction in the ratio of CD8^+^ T cells, while simultaneously decreasing TNF-α and IFN-γ levels and PBMC cytotoxicity and increasing IL-10 and TGF-β levels. In vivo, silencing lncRNA SNHG12 mitigated tumor growth and immune escape, decreased PD-L1 and USP8 expression, and increased PD-L1 ubiquitination level in tumor tissues. Overall, our findings unveiled a novel mechanism in NSCLC development wherein lncRNA SNHG12 promotes immune escape by binding to HuR to increase mRNA stability and expression of PD-L1 and USP8, thus accelerating tumor growth.

## Conclusions

Our study is the first of its kind, as it determined the role of lncRNA SNHG12 as a predictor of prognosis and clinicopathologic features of patients with NSCLC and its ability to promote the immune escape of NSCLC and tumor growth when linked with the HuR/PD-L1/USP8 axis. Collectively, our findings provided insight into the role of lncRNA SNHG12 as a potential therapeutic target in immunotherapy. Nevertheless, with the exception of immune escape, the role of lncRNA SNHG12 in other aspects of NSCLC progression is yet to be thoroughly understood, and the expression pattern of HuR in NSCLC cells remains unclear. Therefore, the functions of lncRNA SNHG12 in other aspects of NSCLC progression, such as metastasis, will continue to be emphasized in future studies to provide an updated theoretical understanding that could aid the finding of NSCLC treatment.

## Data Availability

The data supporting the conclusions of this article are available from the corresponding author on reasonable request.

## Abbreviations

LC: Lung cancer
NSCLC: Non-small cell lung cancer
ICBs: Immune checkpoint blockers
LncRNAs: Long noncoding RNAs
SNHG12: Small nucleolar RNA host gene 12
HuR: Hu antigen
RPD-L1/PD-1: Programmed death-ligand 1/programmed death-1
USP8: Ubiquitin-specific protease 8
FBS: Fetal bovine serum
PBS: Phosphate-buffered saline
CCK-8: Cell Counting Kit-8
PBMCs: Peripheral blood mononuclear cells
qRT-PCR: Quantitative real-time polymerase chain reaction
LDH: Lactate dehydrogenase
FITC: Fluorescein isothiocyanate
PI: Propidium iodide
IFN-γ: Interferon-gamma
TNF: Tumor necrosis factor
TGN: Transforming growth factor
IL: Interleukin
RIP: RNA immunoprecipitation
Co-I: Co-immunoprecipitation
SDS-PAGE: Sodium dodecyl sulfate–polyacrylamide gel electrophoresis
cDNA: Complementary DNA
ANOVA: Analysis of variance
